# High-performance liquid chromatography ultraviolet-photodiode array detection method for aflatoxin B_1_ in cattle feed supplements

**DOI:** 10.14202/vetworld.2017.932-938

**Published:** 2017-08-17

**Authors:** Lazuardi Mochamad, Bambang Hermanto

**Affiliations:** 1 Department of Basic Science, Veterinary Pharmacy Subdivision, Faculty of Veterinary Medicine, Airlangga University, Surabaya, Indonesia; 2 Department of Pharmacology, Medical Faculty Airlangga University, Prof. Dr. Moestopo 47, Pacar Kembang, Surabaya, Indonesia

**Keywords:** feeds supplements, high-performance liquid chromatography photodiode array detector, isocratic methods, mycotoxin

## Abstract

**Aim:**

The objective of the current study is to determine the concentration of aflatoxin B1 using high-performance liquid chromatography (HPLC) with a photodiode array (PDA) detector.

**Materials and Methods:**

Aflatoxin B_1_ certified reference grade from Trilogy Analytical Laboratory dissolved acetonitrile (ACN) at 10 µg/mL was using standard assessment. HPLC instruments such as ultraviolet-PDA detector used a Shimadzu LC-6AD pump with DGU-20A5 degasser, communication module-20A, and PDA detector SPD-M20A with FRC-10A fraction collector. The HPLC was set isocratic method at 354 nm with a reverse-phase ODS C18 column (LiChrospher^®^ 100 RP-18; diameter, 5 µm) under a 20°C controlled column chamber. Rheodyne^®^ sample loops were performed in 20 µL capacities. The mobile phase was performed at fraction 63:26:11 H_2_O: methanol:ACN at pH 6.8. A total of 1 kg of feed contained 10% bread crumbs and 30% concentrated, 40% forage, and 20% soybean dregs were using commercials samples. Samples were extracted by ACN and separated with solid phase extraction ODS 1 mL than elution with mobile phase to collect at drying samples performed. The samples were ready to use after added 1 mL mobile phase than injected into the system of HPLC.

**Results:**

We found that the retention time of aflatoxin B_1_ was approximately 10.858 min. Linearity of 0.01-0.08 µg/mL aflatoxin B_1_ dissolved in mobile phase was obtained at R^2^=0.9. These results demonstrate that these methods can be used to analyze aflatoxin B_1_ and gain 89-99% recovery. The limit of detection of this assay was obtained at 3.5 × 10^−6^ µg/mL.

**Conclusion:**

This method was easy to apply and suitable to analyzing at small concentrations of aflatoxin B_1_ in formulated product of feed cattle.

## Introduction

Aflatoxin is a type of mycotoxin produced by certain molds, mainly *Aspergillus flavus* and *Aspergillus parasiticus*, which are typically found on agricultural products. This type of toxin is known to cause death of animals and humans that have consumed contaminated products. The emergence of fungi which produce this toxin is caused by a lack of safe and wholesome during cutting and storage of livestock products including humidity and temperature of storage spaces.

In early 2015, a heightened risk of aflatoxin poisoning became a concern of the Provincial Government of East Java, Indonesia, due to the increase in small industries which commercially process and market animal feed traditionally without considering sanitation and/or product quality [1].

The most common isoforms of aflatoxin are B_1_, B_2_, G_1_, and G_2_; the B isoforms tend to be more toxic. Aflatoxin B_1_ is produced by both *A. flavus* and *A. parasiticus*, whereas aflatoxin B_2_ is only produced by *A. parasiticus*. When these aflatoxin isoforms enter the body, they split into aflatoxins M_1_ and M_2_, respectively [2].

Aflatoxins can be analyzed by several methods other than immunoassays (e.g., enzyme-linked immunosorbent assay). For example, some studies have used physiochemical methods with optic detector groups such as spectrophotometer ultraviolet-visible (UV-Vis) light, infrared (IR), or adsorption-partition basic system instruments, namely, chromatography groups with gas and liquid mobile phase and also combining with mass spectra [3,4].

That method at above is officially accepted as qualitative and quantitative analytical methods for analysis commonly mycotoxin. High-performance liquid chromatography (HPLC-UV) or fluorescent detector was recommended from Food and Drug Administration to analysis mycotoxin groups although they need confirmation result by other instruments such as Fourier transform-IR, Proton, Nitrogen and Carbon nuclear magnetic resonance, or thermal instrument [4,5]. That method as described above is laborious with skilled personal and usually update method and also instrument to determine the small amount of analyte. They need a person at skillful competence for separation technique of analyte from matrix samples. The analysis of mycotoxin groups sometimes they needed a special column separated to isolation of analyte although that effort not usually success. Some researchers using HPLC fluorescent detector with separate analyte by special column of mycotoxin for isolation aflatoxin groups were thought to be complicated and cost effective [6-8]. Using HPLC-UV photodiode array (PDA) detector with technique preparation by non-specific separate column to determine one of aflatoxin groups as identify B_1_ was not ever applied yet. That was thought to be simple, rapid, and cost effective to implement. In the current study, aflatoxin B_1_ was analyzed using a PDA with UV-Vis light detector together with HPLC. This method is expected to be further developed in laboratories that only have HPLC with UV-Vis light detectors [6,7,9].

## Materials and Methods

### Ethical approval

Using standard Certified Reference Material (CRM) of Aflatoxin B_1_ was under control by Hazardous Agent Control Unit from Indonesia Veterinary Pharmacy and Pharmacology Association (www.affaveti.org) by letter April 3, 2016.

### Feed samples

Cattle feed was obtained from a local cattle breeder in Surabaya, Indonesia; 1 kg of feed contained 10% bread crumbs, 30% concentrated (rice bran 4.5%, coconut cake 3%, pollard/wheat flour 13.5%, fish meal 3%, urea 0.6%, molasses 3%, leather soy 1.5%, salt 0.3%, and vitamin livestock 0.6%), 40% forage, and 20% soybean dregs. An artificial feed free from aflatoxin was used as a blank matrix and was obtained from the Laboratory of Veterinary-Pharmacy, Faculty of Veterinary Medicine, Airlangga University (Surabaya, Indonesia).

The HPLC UV-PDA detector used a Shimadzu LC-6AD pump with DGU-20A5 degasser, communication module-20A, and PDA detector SPD-M20A with FRC-10A fraction collector. The HPLC was set at 354 nm with a reverse-phase ODS C18 column (LiChrospher^®^ 100 RP-18; diameter, 5 µm) under a 20°C controlled column chamber. Rheodyne^®^ sample loops were performed in 2 µL capacities. The certified reference material for aflatoxin B_1_ was obtained from Trilogy Analytical Laboratory, 870 Vossbrink Dr. Washington, MO 63090, USA (cat. no.: TS-104 (P87); lot: 140909-295). All reagents for the mobile phase used were chromatograph grade from Merck Chemical Corp, Germany. The solid-phase extraction (SPE) used a SOLA™ HRP column from Thermo Scientific, USA (serial no.: 19257A1A; capacity, 1 mL). The HPLC was located and run in a room at 22°C and 50% humidity. The injector syringe was obtained from Supelco Corp., USA (analytical capacity, 100 µL).

Aflatoxin B_1_ certified reference material was pulverized to facilitate dissolution in 10 mL acetonitrile (ACN) to make a 25 µg/mL stock solution that was serially diluted into 0.01-1 ppm working solutions. The mobile phase consisted of 63:26:11 H_2_O:methanol:ACN, adjusted to pH 6.8 with glacial acetic acid, and was filtered through a 0.45 µm membrane filter in a vacuum. The mobile phase was then pumped through the column at a flow rate of 0.5 mL/min and 65-67 kgf/cm^2^ pressure. The column equilibration time was about 45 min before analysis was initiated. The active substance was determined by UV-HPLC using an isocratic method with a stop time adjusted to approximately 30 min. Analyses were adjusted with a two-stage process as follows: First step optimization to obtain system suitable test condition by validation processes, then determination of aflatoxin B_1_ at second stage [10].

Method validation was performed to assess selectivity, linearity, intraday precision, accuracy, and sensitivity (i.e., detection and quantification limits) [11]. Selectivity (α) was evaluated by injecting the pure aflatoxin standard and then calculating the retention time chromatogram both of aflatoxin B_1_ with impurities using equation 1 at bellow; acceptable selectivity (α) was defined as α ≠ 1 [12].


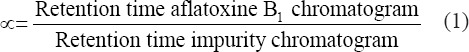


Linearity was assessed with aflatoxin B_1_ stock dilutions (0.2-1.6 ppm) to response detector by correlation regression analyzing; good linearity was defined as a correlation/regression test with an R^2^ value near 1 [13]. Analysis intraday precision was assessed at once period during the physic-chemistry characterization of molecule aflatoxin B_1_ still stable dissolved on solvent. Intraday precision was measured by analysis of aflatoxin B_1_ stock dilutions (0.1-1 ppm) as much as 3 times the area of the chromatogram to obtained percent of coefficient variation (CV); a %CV <3% meant suitable precision was achieved [14]. Accuracy was examined using matrix fodder that did not contain aflatoxin and further purification of aflatoxin using SPE columns then comparing the results to the aflatoxin in the eluent; acceptable accuracy was defined as a recovery with 80-120% CV [15]. Detection and quantification limits (sensitivity) were assessed by first comparing height of chromatograms aflatoxin B_1_ from low concentrations as numerator to height of chromatogram noise as denominator with the requirement referred to equation 2;


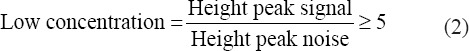


The low concentration of aflatoxin B_1_, then continuously injected to HPLC system for measuring distance of noise peak (Np) - peaks (p) at 20 times the width of the standard peak. The requirement is Np-p (millimeter) not more than 3 mm. Then, we injected a minimum of three low concentrations of aflatoxin into the HPLC and obtained a regression equation (y=mx+b) with slope equal to the sensitivity slope (S). Other equations used for obtaining detection and quantification limits were as follows:


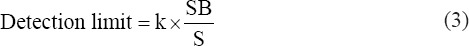



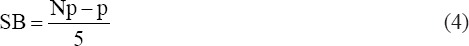


Where, k=3 is constant, SB is the standard deviation of the blank signal, and *S* is the slope or sensitivity slope in Equation 3. In Equation 4, Np−p is the distance highest and lowest peak chromatogram area 20 times the width of the aflatoxin B_1_ chromatogram when the mobile phase is injected into the HPLC [16]. The quantification limit was 3 times the detection limit as described previously [16,17].

Feed samples at 1 g were added 10 mL of ACN than shaked by shaker water bath (27°C) for 60 min than collected in tube 10 mL. The tubes were centrifuged at 1300 g 10 min. Supernatant was pooled in dark bottle than put the dark bottle in the water bath (40°C) and evaporated by nitrogen gas. The dried samples in dark bottle will be kept in room temperature at 22°C as samples ready to use by added 1 mL mobile phase eluent.

Sample preparation required using a triple extraction process to ensure complete separation of pure active substances from impurities in matrix samples. First, the SPE column was activated by adding 1 mL of methanol continuously 1 mL water pro chromatograph, then 1 mL of sample. Second, the SPE column was dried in warm temperature approximately 33-35°C on incubator 30 min before eluting to obtain the active substance (aflatoxin B_1_) chromatogram. The process will occur adsorption-partition mechanism of aflatoxin B_1_ is perfectly SPE column. Finally, the SPE column was eluted with 1 mL of mobile phase, and the eluent containing the active substance (aflatoxin B_1_) was collected in a tube. The eluent containing active compound was then dried with nitrogen gas in a warm incubator (<50°C) as a dried sample ready to use. For determination of aflatoxin B_1_, additional mobile phase was added to dissolve the dried sample, and then the rehydrated eluent was filtered through a 20 µm filter before injecting 20 µL into the Rheodyne^®^ loop.

### Statistical analysis

The correlation regressions among the serial concentration (µg/mL) of aflatoxin B1 to response detector as an area chromatogram were assessed by MINITAB version 17 in 95% confidence interval (CI) with R^2^ analysis.

## Results

**Figure-1:**
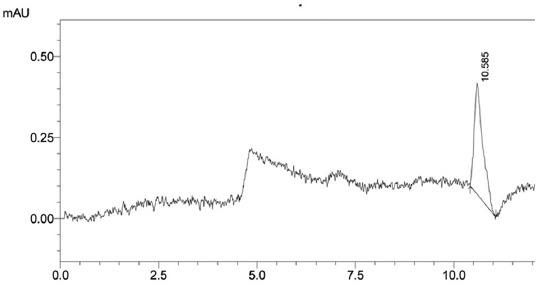
Chromatogram of 0.25 µg/mL aflatoxin B_1_ standard retention time of 10.858 min in the mobile phase (63:26:11 H_2_O:methanol:acetonitrile, pH 6.8) at 354 nm, with a LiChrospher^®^ 100 RP-18 reverse-phase ODS C18 column.

**Figure-2:**
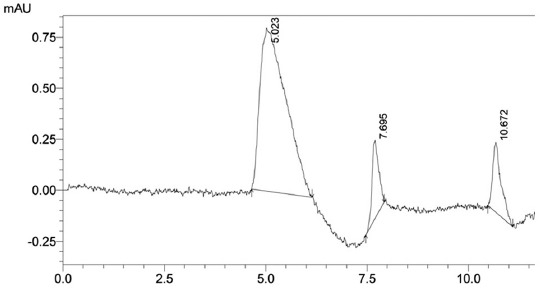
Retention time of aflatoxin B_1_ (10.672 min) in the feed matrix after solid phase extraction column separation from impurities (5.023 and 7.695 min). The α of the first impurity was 2.125 (ratio, numerator 10.672 min to denominator 5.023 min). The α of the second impurity was 1.387 (ratio numerator 10.672 min to denominator 7.695 min).

Figure-1 shows that standard aflatoxin B_1_ in the mobile phase had a retention time of 10.585 min at concentrate 0.25 µg/mL. Spiking the feed matrix with aflatoxin B_1_ produced many impurity peaks and aflatoxin B_1_ peak. Ratio retention time of aflatoxin B_1_ peaks as a numerator versus retention time any impurities peak as a denominator were absolutely ≠ 1. Figure-2 shows that retention time of all impurities peak was not overlay to aflatoxin B_1_ peak. The selectivity criteria (α) showed that aflatoxin B_1_ peak was separated from impurities peak [12]. The data analysis correlations were obtained as follows: 0.24 µg/mL area chromatogram 8,142,042, 0.58 µg/mL area chromatogram 19,676,601, 0.72 µg/mL area chromatogram 24,426,126, and 1.60 µg/mL area chromatogram 54,280,280. Analysis correlation regression between concentration and response detector had a linear perform at R^2^ >0.99 (95% CI, p<0.05).

Intraday precision analysis at serial concentrations 0.17, 0.18, 0.19, 0.54, 0.55, 0.56, 0.74, 0.76, 0.78, 0.94, 0.96, and 0.97 µg/ml shows that % CV intraday precision had not more than 3% (Table 1). Aflatoxin B_1_ in feed matrix after preparation and separation showed 88-98% recovery (Table 2), and detection and quantification limits were 3.5 × 10^−6^ and 1.06 × 10^−5^ µg/mL, respectively [18].

**Table-1:** The intraday precision of aflatoxin B_1_ dissolved in mobile phase.

Concentration (µg/mL)	Area chromatogram
0.18	6,106,531
0.19	6,116,641
0.17	6,006,429
Mean=0.18; CV=5.555%	Mean=6,076,533.667; CV=1.002%
0.55	18,658,846
0.54	17,957,745
0.56	18,758,856
Mean=0.55; CV=1.818%	Mean=18,458,482.33; CV=2.365%
0.78	28,453,985
0.74	24,413,580
0.76	26,433,782
Mean=0.76; CV=2.631%	Mean=26,433,782.33; CV=7.642%
0.94	35,469,650
0.97	38,499,855
0.96	37,488,754
Mean=0.956; CV=1.597%	Mean=37,152,753; CV=4.152%

CV=Coefficient of variation

**Table-2:** Recovery as an accuracy analysis of aflatoxin B_1_ in matrix samples.

Concentration of aflatoxin	Concentration (µg/mL)	Area	Recovery (%)
Aflatoxin in matrix sample			
0.25 ppm	0.200	6,665,032.444	80
	0.220	7,610,343.295	88
	0.240	8,039,522.042	96
Mean±CV	0.22±9.091%	7,438,299.29±9.454%	88±0.022%
0.50 ppm	0.420	14,266,327.55	84
	0.480	16,284,083.8	96
	0.530	17,980,342.55	106
Mean±CV	0.477±11.53%	16,176,917.97±11.494%	95.33±11.55%
1.00 ppm	1.010	34,264,426.55	101
	0.970	32,907,419.55	97
	0.980	33,246,671.30	98
Mean±CV	0.87±2.107%	33,472,839.13±2.11%	98.67±2.11%

CV=Coefficient of variation

**Figure-3:**
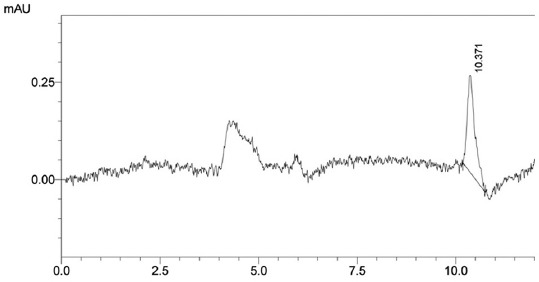
The lowest concentration aflatoxin B_1_ standard (0.05 µg/mL) in mobile phase.

**Figure-4:**
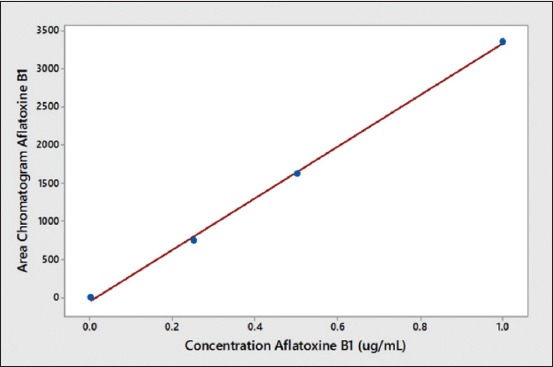
Aflatoxin B_1_ calibration curve in matrix plasma (R^2^=0.99).

**Figure-5:**
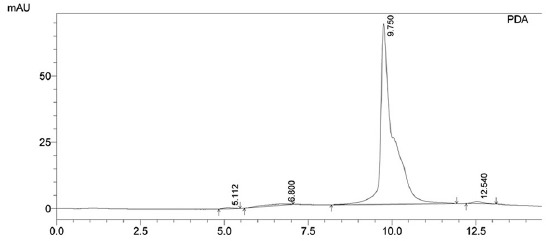
Aflatoxin B_1_ (1.5 ppm) in matrix samples after separated by solid phase extraction showed a retention time of 9.750 min, with an impurities peak at 12.546 min (mobile phase at pH 6.4).

Figure-3 shows the lowest concentration of aflatoxin B_1_ measurable with ratio height of chromatogram aflatoxin B_1_ as a numerator to height of chromatograms impurities as a denominator were ≥5. Figure-4 shows the standard curve of aflatoxin B_1_ in matrix samples with data as follows: 0.0008 µg/mL area chromatogram 55,505, 0.002 µg/mL area chromatogram 1,387,625, and 0.050 µg/mL area chromatogram 34,690,625. The linear regression equation obtained for the lowest aflatoxin concentration was y=−33,925x+0.000246. Using equations 3 and 4 above, the detection limit was calculated as 3.5 × 10^−6^ µg/mL, and the quantification limit was 1.06 × 10^−5^ µ/mL. Figure-5 shows the aflatoxin high concentrate (1.5 µg/mL) at 9.750 min after separated by SPE from samples with impurities peak were found in 12.546 min.

Result research at above was calculated that limit of quantitation for 1 g feed samples dissolved 0.5 mL mobile phase eluent could be detected 2.3 ng of analyte. Comparison to standard method report at worldwide regulations was explained that using liquid chromatography instrument for determining mycotoxin groups in matrix biologic must be detected until 4 ng analyte in 1 g samples [19]. Research result was indicated that method described at above more sensitive than method 2 times at recommended at worldwide regulations.

## Discussion

Our results demonstrate that HPLC UV-PDA detection at 354 and 365 nm can be used to determine the concentration of aflatoxin B_1_ in feed matrix. The peak of analyte (aflatoxin B_1_) was showed easy to identify from impurities peak of other unknown substances in feed matrix. High concentration of analyte was clearly identified if the analyte peak compared to impurities peak. The aflatoxin B_1_ chromatogram at 365 nm was similar to that detected at 354 nm; however, the impurities peak at 354 nm can be minimized more, especially at ranging 1-1.5 µg/mL aflatoxin B_1_.

Isocratic HPLC was suitable for analyzing aflatoxin B_1_ although the retention time usually shifting depend on pH mobile phase. Adjusting the pH range to 6.5 or 6.8 to 7.0 produced the longest retention; 10.672 min. Lowering the pH of the eluent/mobile phase below 6.8 reduced the retention time as shown to research result. No internal standard in that research but we believed that this agent (aflatoxin B1) had a highly potential stable if all processes using chemical with fresh quality condition. Stability retention time of analyte during the adsorption-partition column process was important to identify aflatoxin B_1_ for qualitative and quantitative test. Identification peak of aflatoxine B_1_ from other peaks were used *triplo* or *quadruple* technique injection. First injection to third injection on the HPLC system was identified as samples and following with once injection standard. The retention times of the impurities from the matrix sample after complexion with aflatoxin B_1_ were found at 5.023 and 7.695 min. Sometimes, the other impurity peak was found at a retention time of approximately 12 min, but that peak cannot overly to peak of aflatoxin B_1_.

The α value indicates that retention time of aflatoxin B_1_ relative to that of the impurities had not overlay.

The selectivity assessment (α) was obtained that ratio retention time of aflatoxin B_1_ peak at 10.672 min as a numerator to retention time impurities peaks as denominator at 5.023 min and 7.695 min more than 1, whereas that for the ratio of the aflatoxin B_1_ peak to impurity peak at approximately 12.540 min was <1. The stability compound of aflatoxin B_1_ dissolved in mobile phase was obtained at ranging 12-16 h in condition room 20°C with 50% humidity. Retention time of aflatoxin B_1_ would be shifting less or more than usual 10.672 min depend on external factors, i.e., room temperature and humidity. External factors were known to influence the mobile phase pH [20].

The linearity between aflatoxin concentration and detector response in the mobile phase was found from 0.25 to 1.6 ppm; good linearity at this concentration range was also found in the plasma matrix sample [21,22]. However, determination of less than concentration 0.25 ppm of aflatoxin B_1_ better clarified with other instruments such as liquid chromatography–electrospray ionization mass spectrometry, which can identify the molecular mass of the active substance in matrix samples [23-25].

Studies have shown that HPLC-PDA detection is best when analyzing concentrations ranging from 0.25 to 1 ppm because if we are using a concentration with more than 1 ppm, more impurity peaks will be found every minute [26,27]. Analysis of concentrations <0.2 ppm is recommended with other HPLC instruments’ different detectors, i.e., HPLC with fluorescence detector. Although the theoretical detection limit was 3.5 × 10^−6^ ppm, it must be analyzed by liquid chromatography–electrospray ionization mass spectrometry to confirm [2,28]. As described on Protocol Cartagena that all about biologic products included mycotoxin must be clearly detected by instrument analysis with correlation between physic and chemistry of the compounds [29].

Intraday precision of data showed that our method is good for this type of analysis. The result of intraday precision was that all of the chemical compounds must be *Recenter paratus* [29-32]. We know that aflatoxin was stabilized in the mobile phase or polar eluent for up to 16 h. Unstable aflatoxin was detected at an unstable retention time between 1 and 1.5 min. It is best to use three samples and one standard in the analysis [33,34]. The pure CRM standard of aflatoxin B_1_ shown in demonstrates that quantification to samples for determined aflatoxin B_1_ in feed matrixes requires a concentration ranging from 0.25 to 1 µg/mL.

The findings of the method show that it is very simple and easy to apply, especially in laboratory that only has HPLC PDA detector instrumentation and has limited human resources. Result research was indicated without specific column separated of mycotoxin can be analysis of their mycotoxin. Note that specific separation columns for aflatoxin have limited market availability. That problem above could be dissolved using alternative method from finding this result.

## Conclusion

The current aflatoxin assay using HPLC devices with UV-Vis detectors and a reverse-phase ODS C18 column was isocratic at 354 nm, and the mobile phase consisted of 63:26:11 of H_2_O:methanol:ACN. The stability of aflatoxin in the eluent phase was obtained at between 12 and 16 h at pH 6.8. If the other chromatogram founded at retention times approximately 5, 7, and 12 min was detected, that chromatogram would show the agent of impurity within the matrix samples.

## Authors’ Contributions

LM was a research coordinator and also drafted the manuscript. BH assessed validation method. Both authors read and approved the final manuscript.
